# The Impact of Livelihood Assets on the Food Security of Farmers in Southern Iran during the COVID-19 Pandemic

**DOI:** 10.3390/ijerph18105310

**Published:** 2021-05-17

**Authors:** Masoud Yazdanpanah, Maryam Tajeri Moghadam, Moslem Savari, Tahereh Zobeidi, Stefan Sieber, Katharina Löhr

**Affiliations:** 1 Department of Agricultural Extension and Education, Agricultural Sciences and Natural Resources University of Khuzestan, Khuzestan 6341773637, Iran; yazdanm@asnrukh.ac.ir (M.Y.); moslem_savari@yahoo.com (M.S.); 2 Department of Extension and Rural Development, Faculty of Agriculture, University of Tabriz, Tabriz 5166616471, Iran; m.tajeri67@yahoo.com; 3 Department of Agricultural Extension, Communication and Rural Development, Faculty of Agriculture, University of Zanjan, Zanjan 4537138791, Iran; tahereh.zobeidi@gmail.com; 4 Leibniz Centre for Agricultural Landscape Research, (ZALF e. V.), 15374 Müncheberg, Germany; stefan.sieber@zalf.de; 5 Department of Agricultural Economics, Humboldt University of Berlin, 10099 Berlin, Germany; 6 Division Urban Plant Ecophysiology, Humboldt University of Berlin, 14195 Berlin, Germany

**Keywords:** food security, livelihoods, rural households, COVID-19

## Abstract

The impact of COVID-19 on farmers’ livelihoods and food security is a key concern in rural communities. This study investigates the impacts of the livelihood assets on the food security of rural households during the COVID-19 pandemic and determines those factors related to food security. The population of this study includes rural households in Dashtestan county, Bushehr province, in southern Iran. Based on the Krejcie and Morgan sampling table, 293 households were selected using the convenience sampling method. To measure food security, the American standard index and ordinal regression are used to analyze the factors. The results of the food security situation show highly precarious and food insecure situations among the studied rural households. The regression analysis shows that the most important assets affecting the food security of rural households under COVID-19 are financial, psychological, physical, and human assets, respectively. The results can help rural development planners and policymakers to improve both livelihoods and food security in rural communities, not just during the COVID-19 pandemic, but also in its aftermath.

## 1. Introduction

Globally, the COVID-19 crisis is primarily viewed as an unprecedented public health challenge. While it is not as deadly as the H1N1 flu epidemic, it is unprecedented in the rapid transmission of viral agents from one human to another worldwide. However, it is profoundly and widely affecting socio-economic activity, work life, food systems, and many other sectors. Thus, the pandemic’s effects go far beyond just public health [1,2] as it has wiped out or disrupted various jobs, and as of December 2020, put almost half of the world’s 3.3 billion workforce at risk of losing their livelihoods. Potentially, many breadwinners will lose their jobs, and in the worst scenario, get sick and die [3]. The World Food Programme warns that the world is facing an “epidemic of hunger.” In addition to the 135 million people who were food insecure before the COVID-19 crisis, up to 130 million (nearly double) more people may face acute food insecurity by the end of 2020 [3]. The COVID-19 pandemic can lead to a psychological, economic, and partly physical disruption to markets, social subsystems, and citizens [4]. The potential economic and social turmoil caused by the COVID-19 pandemic could be devastating.

Most measures undertaken by governments to control COVID-19 have affected the livelihoods and the food security of communities [5]. Border closures, quarantines, social distancing, curfews, and trade restrictions prevent farmers from accessing farms and markets—including the purchase of inputs and the sale of their products. Controls also prevent workers from harvesting agricultural products, triggering significant socio-economic consequences for people’s livelihoods [3]. While these restrictions are crucial for limiting the spread of the disease, they often disrupt chain markets and trade in agricultural and non-agricultural products, thus affecting the nutrition and food security of all, with particular consequences for those who are forced to travel for their livelihood [3]. In particular, rural residents and farmers in developing countries are more vulnerable because most of them lack, in their local community, access to resources including clean water, schools, health centers, transportation, communication facilities, and social support, all of which are typically readily available in urban areas. The lack of these resources, services, and support put these populations at a higher risk and vulnerability [3]. In this regard, Tajeri Moghadam et al. [6] point out that residents of rural areas are more vulnerable to the prevalence of COVID-19 than residents of urban areas because hospitals and information centers designated for COVID-19 disease are in urban areas. Rural access to medical centers is difficult due to distance and travel costs; thus, there are barriers to prevention and treatment, resulting in a higher vulnerability of the COVID-19 disease.

In Iran, the COVID-19 pandemic was officially confirmed on 18 February 2020. On 19 February 2020, the Ministry of Health announced that the results of the initial testing of two suspected cases of COVID-19 in Qom were positive. The number of people with COVID-19 and the number of deaths was increasing day-by-day [7].

It follows that individuals who were previously vulnerable (e.g., farmers living in poverty) appear to be disproportionately affected by COVID-19 [8]. Thus, the COVID-19 shock highlights existing vulnerabilities and creates an additional layer of complexity to farmers’ livelihoods and food security [9]. In other words, due to the long history of being affected by shocks, most rural communities and farmers are inherently more vulnerable to future shocks [10] and facing multiple shocks at once [11].

COVID-19 affects the food industry and the food supply chain into four main domains. As consumers seek to protect themselves and their immune systems through healthy diets, the availability of bioactive food and functional foods may become critical as demand for these products increase. Second, food safety is an important issue in preventing the spread of the virus among producers, retailers, and consumers [12,13]. Third, food security issues have arisen due to the containment of one billion people to their homes. Last, the sustainability of food systems in the COVID-19 pandemic era is another issue that this section should consider to avoid or decrease the frequency of relevant food and health crises in the future [12]. For example, sustaining food production during COVID-19 potentially triggers the clustering of cases in agricultural food production, slaughterhouses, and food processing industries [14].

The breakdown of supply chains due to virus contamination and a variety of political constraints pushed up prices, and simultaneously, increased producer costs, thus increasing food insecurity for urban and rural poor [2,15]. Global food insecurity warnings were issued as a result of food shortages, rising prices, and/or loss of income due to rising unemployment [15]. The COVID-19 pandemic rapidly affected the entire food system, revealing its fragility as it undermined food security both directly by disrupting food systems and indirectly by the impacts of quarantine on household incomes and physical access to food [3,8,16]. For most people, not having productive assets and income means, in the worst case scenario, not having food; in the best scenario, there is less food than is typically nutritionally unbalanced [3].

Food insecurity is a stressor. Stress associated with the COVID-19 pandemic revolves not just around where and how to access food, but potentially also employment, financial hardship, livelihoods, disconnections from social support systems, and worrying about the health of oneself and loved ones [17]. To implement appropriate policies that reduce food insecurity, it is important to understand the impact of livelihood assets on food security and to investigate the relationship between livelihood assets and food security to achieve sustainable development paths. Further, a full understanding of the impacts of pandemics on household livelihoods requires investigating their impact on household assets [18]. There is ample evidence in the literature that epidemics, like HIV, Ebola, and malaria, profoundly affect the livelihoods of individuals, families, and communities [19]. However, there is not yet a study investigating the impact of rural household livelihoods on food security during COVID-19. This study investigates the impacts of livelihood assets on the food security of rural households during the COVID-19 pandemic and determines those factors related to food security. To analyze factors affecting food security, the framework of livelihood assets, including different capital, is considered. In this study, unlike existing studies that use dummy variables or changes in household income to assess the impact of shocks [20], the impact of different livelihood assets on food security is examined. Therefore, using advanced methodological innovations, findings from this study can help policymakers plan interventions for livelihoods that are susceptible to pandemics. The study hypothesizes that food security during COVID-19 is strongly dependent on livelihood assets Therefore, the Sustainable Livelihoods Framework is applied to elucidate the context of vulnerability (here the COVID-19 shock) and farmers’ access to livelihood assets. Despite the importance of other livelihood goals, the impact of COVID-19 on rural household’s food security is our concern. This study evaluates the relationship between livelihood assets and food security during the COVID-19 pandemic in rural areas of Dashtestan county, Bushehr province, southern Iran. The results of this assessment help to minimize farmers’ vulnerabilities during the pandemic. Furthermore, the role of this study is to inform policymakers by identifying groups at risk of food insecurity.

## 2. Research Framework

In this study, we use parts of the sustainable livelihoods framework (SLF) developed by Chambers and Conway [21], and described by others [22,23]. In the sustainable rural livelihood approach, the main goal is to rely on the main assets and capital (human, social, financial, natural, and physical) in the rural area as the primary and basic sources of rural livelihoods. The sustainable livelihoods framework incorporates research on poverty reduction, sustainability, and livelihood strategies, and sustainable livelihoods defined as “Livelihoods include facilities, assets (inventories, resources, receivables, and access), and activities needed for living. A livelihood is sustainable when it can overcome stress and shock or it can recover from these stresses and shocks, or when it maintains or enhances its capabilities and assets, provide sustainable livelihood opportunities for future generations” [21] (pp. 7–8).

The livelihood strategies that people adopt depend on their ability to access, defend, and maintain a wide range of assets (also referred to as resources or capital) [23]. These assets are classified as natural, social, human, physical, and financial capital. These assets play an important role in survival strategies for rural and urban livelihoods [24]. Instead of focusing on one particular asset separately, this framework recognizes that assets are combined to pursue strategies, like livelihood diversification, which can either produce food directly or provide an entitlement to it. An essential aspect of a sustainable livelihood framework is the role played by the context of vulnerability (here, COVID-19 threats). This includes context of vulnerabilities or shocks experienced over time [22].

### 2.1. Conceptualization and Food Security Status of Households

Food security is inherently complex and is a principle risk factor for individual and social health that is essential for the sustainable development of society [25,26]. Food security means always providing all people with access to healthy and adequate food, through socially acceptable methods, in order to have a healthy life [27].

In the opposite situation, food insecurity can be described as limited or unsafe access to adequate and nutritionally safe food or limited ability, if not an inability, to obtain acceptable foods through community-acceptable ways [26].

Given that food security is an indicator of family and individual health, it can be a precursor to health and nutritional problems. Food insecurity can be chronic, seasonal, or transient, ranging from anxiety about access to food at the household level to severe hunger in children [25]. Food insecurity is a global concern due to the increasing number of people who remain undernourished, amounting to 842 million individuals, approximately 12% of the world’s population [27]. In Iran, statistics indicate worsening food insecurity among Iranian households in rural regions: the study of Pakravan Charvadeh et al. [26] shows that the food insecurity situation is much more severe in rural Iran, with almost one-third (32.4%) of the rural population facing food insecurity. In general, food security improvement policies should target rural areas with the highest percentage of food insecure households [26].

While the COVID-19 is a public health disaster, there are concerns about its potential consequences for local and global food systems, including its capacity to ensure access to healthy and affordable food, as well as adequate income for low-income people, especially, smallholder farmers in developing countries [28]. COVID-19 affects six pillars of food security:

- Availability: Quarantine and restrictions on the movement of people affect farmers’ access to farms and agricultural activities. If farmers have trouble accessing their farms, this may eventually lead to less production, subsequently affecting food security, not just now, but also in the future.
- Access: COVID-19 conditions in different countries are reducing people’s purchasing power. In countries like Afghanistan, where about half the population lives in food insecurity, the COVID-19 pandemic was disastrous, reducing purchasing power. Restrictions on transportation and closure are serious challenges for maintaining secure trade throughout the rural economy in multiple countries [5].
- Utilization: The loss of purchasing power, especially for the poor, including daily wage workers and small business families, led to changes in people’s consumption patterns, and consequently, poorer nutrition. In Uganda, communities survive on one meal a day. It is also difficult to produce fresh agricultural products in some areas. In many countries, it is difficult to prepare fresh vegetables. In this period, when people think they can build their immunity (also against COVID-19) with proper nutrition, they are unable to buy food due to a lack of funds, and in many cases, even if they have money, food availability is limited.
- Stability: During COVID-19, food storage is a daunting challenge in many countries and sometimes difficult to achieve [5].
- Agency: During COVID-19, disadvantaged individuals and communities, including women, smallholder farmers, and vulnerable workers, were unable to act independently to make choices about what they eat, the foods they produce, how they are produced, processed and distributed, as well as their involvement in the policy processes that shape food systems [29,30].
- Sustainability: The COVID-19 pandemic is an alarm for thinking about supply chains and resilience of future food systems. During this period, many issues, like nutrition and food sustainability, along with the need to take into account the long-term developments resulting from slow economic recovery, changes in consumer behavior, and disruption to risk management should be reconsidered [29,30,31]. A study by Pakravan-Charvadeh et al. [32] on the short-term effects of the prevalence of COVID-19 on Iranian households’ food security shows that the food security of Iranian households that had food security before the pandemic of COVID-19 improved during the early period of the pandemic. Compared to the time before the pandemic, households reduced their intake of specific food sets (vegetables) throughout the pandemic. During this period, the percentage of households facing severe food insecurity decreased from 21% to 17%. Socio-economic causes related to food insecurity in the pandemic period also include household income, personal savings, employment status, and nutritional knowledge of the head of household. Nutritional knowledge is the most important factor in improving food security during a pandemic. In Jordan, Elsahoryi et al. [33] found that, during COVID-19, almost all individuals are concerned about shortages and the inability to prepare staple foods.

### 2.2. Livelihood Assets, Resources and Capital

The turbulent situation in the wake of COVID-19 highlights the need for access to livelihoods [34]. In developing countries, people make their livelihood from a set of assets and capital that typically make clear how they earn their livelihood by simple inspection [35]. Assets are important to the poor because they can help them better cope with shocks, including climate shocks, and the long-term effects of severe weather and infectious diseases. In investigating ways out of poverty for poor rural people, research on asset-based approaches to poverty reduction since the 1990s shows that asset control plays a key role in increasing income, reducing vulnerability, and empowering people, and thus it provides the ultimate way out of poverty [36].

Assets are resources that people have access to, comprising private goods (household capital) and/or public goods (community capital). Family assets are classified into a set of five subsistence assets: natural, physical, financial, human, and social [18,24]. These assets play an important role in survival strategies in sustainable rural and urban livelihoods [24]. It is the combination of these assets that provides adequate and sustainable living conditions for humans [34].

Natural assets are the natural properties that individuals rely on for their subsistence and progress. As the most significant natural properties of farmers, land and water play a vital role in the livelihood of rural families [37,38]. Physical property typically refers to basic services and infrastructure, such as roads, water supply canals, production tools, and equipment (tractors), which facilitate farmers’ production and livelihoods. The total value of agricultural machinery and equipment reflects the physical assets of farmers to produce agricultural products, which supports increasing the effectiveness of agricultural production. Financial assets mainly refer to the total quantity of cash accessible to the public and may also include access to credit and loans. Human property is largely associated to knowledge, skills, health, and the ability to work. Social property, as a network of social relationships between individuals or groups, is considered to be those social resources that individuals use to help their livelihoods. Farmers often share their capabilities and knowledge about agricultural practices through face-to-face communication with friends and relatives. In addition, the level of trust among neighbors is beneficial for creating a good situation for communication and interaction, thus sharing experiences of agricultural production in rural areas [37,39].

Studies show that not all shocks are expected to have the same effect on livelihood assets and outcomes. The study by Chiwaula and Waibel [20] on a fishing community in Nigeria shows that people in a village are affected differently by different shocks due to differences in their capital assets and livelihood activities. Some studies examine only one dimension of livelihood assets. For example, Mbiba et al. [40] argue that rural households with limited access or a lack of access to natural resources often have difficulty in obtaining food, amassing other assets, and recovering from natural or market shocks. There are also studies on the impact of shocks on social capital. For example, Berhanu’s [41] study shows that shocks through poverty traps significantly erode trust and confidence in traditional social support systems and increase dependence on ancillary agencies. Gatiso et al. [19] pointed out that shocks like epidemics weaken some or all of these five family assets and negatively affect livelihoods. The results of their study show that the prevalence of Ebola in the community negatively affects the production of household crops, which may exacerbate the problem of food insecurity throughout Liberia. In addition, they find that the Ebola epidemic undermined public confidence in Liberian institutions. A study by Ansell et al. [42] showed that AIDS contributes to food insecurity in South Africa and negatively affects the access of some rural youth to livelihood assets.

**Figure 1:**
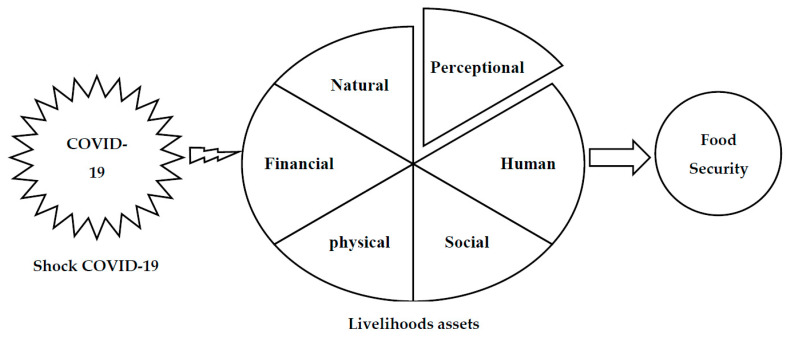
Research framework.

Many researchers in the realm of shocks [43,44,45] argue that the response to threats is a two-step process, with understanding the risk (perception) comprising the first step and responding to it the second. Based on this argument, Shinbrot et al. [46] include perception as a new asset in the current livelihood assets framework. Thus, inspired by them, we use perception as a new asset in the framework (see Figure 1). According to various studies, the COVID-19 shock, as a context of vulnerability, affects farmers’ livelihood assets and food security.

## 3. Materials and Methods

### 3.1. Study Design

The main purpose of this study is to investigate the effect of livelihood assets on food security of rural households in COVID-19 conditions.

### 3.2. Sampling and Data Collection

This is a cross-sectional survey with data collection occurring in May 2020. The population of this study comprises farming household heads in rural areas in Dashtestan county, Bushehr province, in southern Iran (*n* = 19,812). This study investigates the impacts of livelihood assets on the food security of rural households under COVID-19 conditions in Iran, as it is the country with the highest prevalence and spread of COVID-19 in the Middle East. At the time of data collection, in May 2020, the Iranian Ministry of Health announced that the official statistics of the total number of patients in Iran had reached 175,927 and the total number of deaths reached 8425. In our study area, the number of COVID-19 patients in Bushehr province at the time of data collection was 2100, of which 428 are from Dashtestan County. This situation is worse in rural areas than in urban areas due to widespread dispersion and the lack of proper infrastructure. Additionally, Iran is a special case due to its political isolation, though the government proactively took steps to contain the spread of COVID-19. Dashtestan County is well suited to study the impacts of livelihood assets on food security during COVID-19 because its outbreak in March 2020 coincided with harvesting season, resulting in increased vulnerability of farmers. Most farmers produce vegetables or horticulture and reported problems harvesting and marketing products during the first wave of COVID-19. Thus, farmers in the province were severely affected by the damage caused by the COVID-19 pandemic with its related restrictions on mobility and trade [47].

Based on the Krejcie and Morgan [48] sampling table, household heads were selected from different rural parts of Dashestan County. For the purposes of this study, no personal data is collected and stored that could be traced back to individuals. The sampling for the survey takes place at the household level. In this study, two methods were employed to collect data: (1) an online survey using an internet-based questionnaire (*n* = 197) and (2) an offline survey using a paper-based questionnaire (*n* = 96). In fact, to avoid unnecessary encounters during COVID-19, and in order to increase the response rate and to reduce the possibility of sampling bias, two questionnaires based on the Internet and print was designed. The Internet-based questionnaire consisted of a letter and a URL. The printed questionnaire was designed for people who did not have access to the Internet via mobile phones, or laptops. To be sure about the samples, at the beginning of each questionnaire, respondents were asked to complete the questionnaire only if they were the head of the household and their occupation was related to agriculture. Since data collection coincided with the widespread prevalence of COVID-19, and due to the consequent lockdowns, sampling was performed using nonprobability sampling of convenience sampling. Convenience sampling is a type of non-probability sampling and a method under which researchers easily collect research data from an accessible group that is willing to participate. In this study, data were collected wherever sample access was available, such as a center for buying and selling agricultural products, farms, and homes. The personal identification of interviewees is not necessary and does not take place within the scope of the sampling. The questionnaire and its contents are designed in such a way that they do not allow any conclusions to be drawn about individuals. Participation in the study is voluntary and takes place on the basis of informed consent. No written form of consent is used, but all interviewees are informed about data protection issues by the enumerators and asked to give their consent orally at the beginning of each interview.

### 3.3. Instrument

This research was generally conducted in two steps. In the first step, the food security status of rural households during the COVID-19 period (over a period of six months) was assessed. During these six months, rural households were asked about the occurrence of food security items (18 food security items) in their life. The statements were organized in three groups (never, sometimes, and often).

In order to investigate the food security situation of rural households, the standard 18-item questionnaire of the US Department of Agriculture (USDA) is used. This questionnaire is frequently used in developing countries due to its consideration of all aspects of food security. Additionally, it has been used in rural Iran before, where its reliability and validity are confirmed [15]. For the purpose of this study, households with children and without children were separated, because eight items out of 18 items in the USDA Standard Food Safety Questionnaire are for children, while households without children were not asked for these eight items and were not used as a basis for grouping. Therefore, for households with children, 18 items were used, and for households without children, 10 items were used based on the range of never (as a negative answer to the question), sometimes and often (as a positive answer) in order to measure food security.

Coefficient of variation was used to prioritize the 18 Food Security Items, which is a normed criterion used for measuring the distribution of statistical data calculated as Sd divided by the mean as the following:

CV = Sd/µ(1)

In other words, it demonstrates the dispersion rate per as a unit of the mean condition for the mean not to be zero [49].

In order to group the food security situation of rural households, the standard grouping of the USDA is used [50]. Rural households were grouped based on a positive response to (10 or 18) food security items (Table 1). If a household score is less than 2.32, it is considered as food secure; if scoring more, then the household faces food insecurity. Food insecurity itself is divided into two parts: food insecurity without hunger (household score between 2.32 and 4.56) and food insecurity with hunger (household score greater than 4.56), which is subsequently divided into two further parts. It is divided into food insecurity with moderate hunger (household score 4.56 to 6.53) and food insecurity with severe hunger (household score more than 6.53). Items were weighted to obtain the values in Table 1, according to the world standard.

In the second step, the effects of the COVID-19 pandemic on livelihoods assets were assessed based on the framework by Pakravan-Charvadeh et al. [51]. Here, livelihood assets include physical, financial, human, natural and social capital; however, according to the literature review, the perception one of the important assets during the COVID-19 pandemic was added to livelihood capital (Figure 1). The items in this section were measured based on the Likert scale (1- very low to 5- very high) (Table 2). Finally, for classification and grouping effects of COVID-19 on livelihood assets, the criterion of difference of standard deviation from the average (ISDM) was utilized as follows: [52].

(2) $$\mathrm{Low}:\;A\;<\;\mathrm{Mean}-\frac{1}{2}\mathrm{Sd}\;\;\mathrm{Medium}:\;\mathrm{Mean}-\frac{1}{2}\mathrm{Sd}\;<\;B\;<\;\mathrm{Mean}+\frac{1}{2}\;\mathrm{High}:\;C\;>\;\mathrm{Mean}+\frac{1}{2}\mathrm{Sd}$$

It should be noted that, in the formula above, A, B, and C shows the level of COVID-19 impacts on livelihood assets with A being low, B representing medium, and C showing a high level. The mean in the formula indicates the ordinary mean or arithmetic mean, and is Sd considered as one of dispersion indicators [53]. ISDM measure stands for Integrated Science Data Management, used when the author intends to classify or group a special subject [49]. According to the ISDM standard, each indicator level (such as COVID-19 impacts on livelihood assets) is determined with respect to the distance of the indicator in question from the mean and standard deviation of the same indicator in the population [54]. In general, the most important reasons for using ISDM in this study are: (1) identification of the effects of COVID-19 in lower classes in time for better decision making by policymakers because respondents are categorized and their vulnerability is determined; and (2) it was used in statistical analysis to measure the effects of COVID-19 on food groups.

In step 3, after identifying the effects of COVID-19 on food security and livelihood assets, the effects of livelihood assets on food security are investigated through Chi-square tests and ordinal regression.

### 3.4. Validity and Reliability of Instrument

To check the overall indicators before interviewing the farmers, the draft survey and questions were reviewed by an expert board. The expert panel included professors in the fields of agricultural extension and education, food security, environment, psychology, social sciences, and agricultural sciences. Adaptations were made until final approval on the basis of their views was achieved. In addition, Cronbach’s alpha coefficient test was used, the value of which was between 0.70 and 0.95 for different constructs of the questionnaire. Therefore, the research tool has acceptable validity and reliability.

### 3.5. Data Analysis

To perform data analysis for both the descriptive and inferential sectors, SPSSwin18 was used. To simultaneously distribute the effects of the COVID-19 on food security and livelihood assets, the Chi-squared test was applied. It is a statistical test used to assess the interrelations of nominal variables [55]. The Chi-squared test is a valid statistical test that can be used to study the systematic relations of two variables [56]. This test is usually employed for relations in which both variables are non-parametric [55]. The test shows if there is a statistically significant difference between the frequency of the observations and the expected frequency of one or more groups of a contingency table. The contingency table in the present work was formed between the levels of COVID-19 impacts on livelihood assets (financial, psychological, physical, natural, human, and social) with the status of food security (food secure, food insecurity without hunger, food insecurity with moderate hunger, and food insecurity with severe hunger).

Since the criterion variable (food security) was a stratified ordinal variable, ordinal regression was employed. The ordinal regression that is based on the McCullagh methodology is known as the ordinal logistic regression [57]. The ordinal regression allows for modeling the dependence of an ordinal dependent variable on a series of independent variables [58]. One goal of ordinal regression is to optimize the response variable in different problems. This means that, with a change in the status of controlling variables, an optimal status is obtained from the response variable [59]. In this type of regression, the regression coefficients show how changes in the independent variables influence the dependent variable [60]. This research also explored the impact of livelihood assets as independent variables on the dependent variable (food security).

## 4. Results

### 4.1. Descriptive Statistics

Based on demographics results, the respondents’ average is 43.27 years old. The mean family size and years of experience in farming are 3.84 people and 9.73 years, respectively.

### 4.2. The Situation of Households’ Food Security under COVID-19 Condition

As described in the methodology section, the standard 18-item questionnaire of the USDA is used to investigate the food security status of rural households during the COVID-19 pandemic. The questionnaire considers the temporal process, facilitating the analysis of food security status. As mentioned in the research method, the coefficient of variation was used to prioritize food security items among rural households during the COVID-19 pandemic. The prioritization exercise shows that the items “could not afford to eat balanced meals” and “worried food would run out” are the main food security problems of rural people during the pandemic (Table 3).

**Figure 2:**
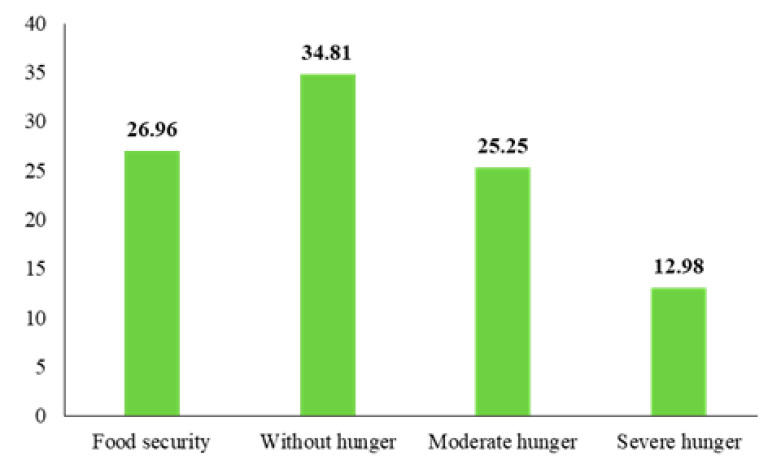
The situation analysis of households’ food security.

Using the USDA scale to group households based on their food security, shows that 26.96% of the studied rural households were food secure, 34.81% were food insecure without hunger, 25.25% were food insecure with moderate hunger, and 12.98% were food insecure with severe hunger (Table 1). Overall, the vast majority, 73.04%, do not have food security (Figure 2).

### 4.3. The Impacts of COVID-19 on Livelihood Assets

Status livelihood assets are ranked by the coefficient of variations. The results in Table 4 show that the greatest impact of COVID-19 was on the financial and psychological assets of rural households. However, in general, based on the results, it could be declared that 34.81% of the households have faced lower impacts of COVID-19, relatively. 41.29% of them have faced moderate impacts, and 23.9% of the households have faced higher impacts than other groups.

### 4.4. Investigating the Relationship between Livelihood Assets and Food Security

The distribution of the livelihood assets and food security of rural households is checked using the chi-squared test (Table 5). The results show that the comparison of the frequencies of the livelihood resources based on the food groups is 1.082, which is significant at the 0.01 level. Therefore, there is a significant difference between livelihood assets and food security levels. Based on the results in the contingency table, it can be said that the less influential that COVID-19 is on the livelihood resources, the higher the food security of the rural families will be because the food security groups were placed at the levels of the contingency table in which COVID-19 was less influential on livelihood assets.

### 4.5. Impacts of Livelihood Assets on Food Security of Rural Households under COVID-19 Conditions

The predictive factors of the food security levels are identified by ordinal regression (Table 6 and Table 7). Since the chi-square statistic is significant, it implies that the data has a good fit and could well explain the probability of the variations of the dependent variable (Table 6).

Table 7 presents the findings as to the fit of the ordinal regression model for the effective assets influencing the rural households’ food security levels under pandemic conditions. To understand the significance of the inclusion of each independent variable in the model, the Wald statistic is employed, which is equivalent to the t-statistic in linear regressions. Based on the results, the regression of four livelihood assets (financial, psychological, physical and human) have a significance level of <0.05, thus showing that their inclusion in the model is useful. That is, only these four livelihood assets have a significant effect on improving food security. Subsequently, the estimation by the ordinal regression model indicates the share of each independent variable in capturing the likelihood of changes in the size of the food security level.

Y = 0.076 (FIA) + 0.068 (PSA) + 0.055 (PHA) + 0.058 (HUA)(3)

As mentioned in the materials and methods, in order to determine the importance of the effect of independent variables on the dependent variable, the estimation coefficient should be considered. Based on Equation (3), financial (0.076), psychological (0.068), human (0.058), and physical (0.055) factors have the greatest effects on the food security of rural households under COVID-19 conditions.

In ordinal regression, it is important to check the proportional odds assumption of different levels of the dependent variable, which is done by the test of parallel lines (Table 8). Based on the results, the significance of the chi-square statistic is >0.05, supporting the null hypothesis of the proportional odds assumption among the levels of the dependent variable.

## 5. Discussion

This study investigates the relationship between livelihood assets and food security during the COVID-19 pandemic in southern Iran. This is the first investigation into the impact of the livelihood assets on food security of rural Iranian households. We found that the studied community is not receiving optimal nutrition during the pandemic. Before the pandemic, approximately 32% of Iranian families were food insecure [61]. With the persistence of the COVID-19 pandemic, the Iranian government adopted policies designed to cope with its effects, including extensive lockdowns, social distancing, quarantines, traffic restrictions, and commercial restrictions. Combined, these policies affect the livelihood and food security of rural families, which were already vulnerable. Indeed, during the quarantine and restriction period, most rural families were unable to provide food for themselves due to the constraints on their livelihood assets and their income instruments. Further, the lockdown of roads and restriction of travel not only limited food transportation and distribution, but also reduced food production, availability, and consumption while simultaneously increasing food prices [7]. All these activities and policies created poor food security in the study area. This finding is consistent with the results of other studies [33,62,63,64] with respect to the status of food security during the COVID-19 pandemic in other countries. International organizations (FAO, WFP, and UN) have also predicted and warned that the COVID-19 crisis is a threat to the livelihoods and food security of vulnerable people. However, it is inconsistent with the results of Pakravan-Charvadeh et al. [32] regarding the short-term impacts of the COVID-19 outbreak on food diversity and food security status among urban families of Tehran province, which was conducted during the first wave of the outbreak. They report an improvement in food security during the first wave of the outbreak. We believe that the underlying reasons for this inconsistency are rooted in the differences in the studied populations (urban families in Tehran province vs. vulnerable rural families in the south of Iran) and data collection time (at the beginning of the COVID-19 outbreak versus the second wave of COVID-19 outbreak).

Our results on the food security of the studied population show that the main problems confronting rural families during the pandemic conditions are “could not afford to eat balanced meals” and “worried food would run out,” which is consistent with Elsahoryi et al. [33] and Chiwona-Karltun et al. [65]. Under COVID-19 conditions, most rural families are faced with problems of supplying appropriate food for their family members due to the quarantine, the restriction policies, and the inflation of food prices. Higher food prices along with the reduced income of rural families imply that most households must reduce the quantity and quality of their food regime, which can potentially have long-term impacts on their nutrition and health. However, due to the panic over the depletion of essential commodities and foodstuffs, some people who were in a better place, in terms of finances, purchased commodities in large quantities and hoarded them. In these conditions, the most vulnerable rural families worry about fully depleting their meager pantries. Given the poor status of food security in the studied community and the fact that people require even better access to nutritious and adequate food during the pandemic in order to strengthen their immune system, it is necessary for planners and policymakers to consider improving the food security of rural farmers. The impressive effect of food supplements and nutrients is proven to be a possible prevention against COVID-19 and supporting the immune system. These nutrients will also help consumers protect themselves during the post-lockdown recovery. Therefore, there is an urgent need for widespread access to healthy foods, and people should be aware that healthy eating habits may reduce the sensitivity and long-term effects of COVID-19 [66]. To improve the food security of rural people, it is necessary to provide them with supportive subsidies, livelihood packages, gratuitous or low-interest loans and credit facilities, guaranteed purchase of their crops, governmental surveillance of food production and the distribution chain, attention to the fair distribution of services and facilities, avoidance of discrimination in service and facility delivery, as well as even closer surveillance of the prices of essential foods along with immediate adoption of crop price control policies.

Given the transit restrictions, the Iranian government should take action to ensure that crops are purchased at appropriate, fair prices and are marketed in a timely manner. As such, the farmers’ revenue for food supply is preserved and the produced food is provided to people who need it.

In addition to the policies and activities that the government should implement to improve the food security of rural families, non-governmental organizations, charities, and even more prosperous people should also help to improve the livelihoods and food security of rural households by providing them with livelihood assistance packages. In addition, the finding of poor food security reflects the fact that, generally speaking, the policies adopted to improve food security are neither efficient nor effective. These should be revised in a way that increases the food security of vulnerable rural people during pandemic conditions. At the same time, people must be informed about the fact that foodstuffs are available in adequate quantities and they do not need to hoard them. As such, foodstuffs can be supplied to rural families in adequate quantities and at proper prices.

In rural areas, the livelihood of most families depends on agricultural activities. The COVID-19 outbreak is adversely affecting the economic livelihoods and revenue of families. The preventive policies and activities against COVID-19 resulted in the loss of employment and the decline of revenue and available money for rural families, meaning that they face problems in meeting some of their essential needs. For instance, smallholders lost access to markets to sell their products. As a result, they cannot sell their products and this results in food supply declining and prices increasing [67]. In this circumstance, some people may even have to sell their assets to meet their essential needs, which may jeopardize their long-term economic livelihood.

Following financial assets, the psychological assets of the families are most deeply affected by the COVID-19 pandemic. Most rural families are in poor conditions in terms of health facilities, with most of the studied villages having no clinic or health care center. Families even lack access to basic health facilities, including disinfectants (e.g., alcohol), face masks, gloves, and so on. Further, most people suffer from severe stress as a result of the pandemic, including the high mortality rate of COVID-19, the imposed social distancing, depression caused by isolation, as well as the chronic stress and anxiety for economic problems resulting from it [68]. Consequently, COVID-19 has had adverse impacts on the mental health status of rural families, significantly reducing their social vitality.

Regarding the distribution of the relationship between the livelihood assets and the food security of the studied rural households, there is a significant difference between the impacts of the pandemic on livelihood resources and its impacts on food security levels. The severe impact of livelihood assets results in severe food insecurity, its moderate impact results in moderate food insecurity, and, finally, its low impact results in mild food insecurity. Most studies [7,9,41] report that, due to the impact of the epidemic and restriction policies on crop production, income, transportation, and food chains, most rural families are losing some of their livelihood assets, which not only reduces their wellbeing, but also aggravates the problem of access to foodstuffs and overall food insecurity.

Based on the results of the ordinal regression, the most important assets affecting the food security levels of rural families during the COVID-19 pandemic are financial, psychological, physical, and human assets, respectively. If the livelihood assets of rural families are changed by the COVID-19 shock, then their food security is affected. Under these conditions, the main asset that these families have at their disposal is their financial resources, including available money and credits. Crop production is the primary source of revenue for most rural families in developing countries, but restrictive policies inhibit these income-generating agricultural activities, thereby impairing farmers’ revenue and increasing food insecurity [69].

In these conditions, since financial assets are the most important assets affecting the food security of the rural families and since current conditions show that the income and available money of rural families have declined, it is recommended that the government, non-governmental charities, and prosperous people provide support to farmers who are the backbone of the economy. By doing so, it will help famers to attain food security.

The role of this study is to inform policymakers that groups are at risk of food insecurity. In fact, this study could help improve policies to prevent food insecurity. Although this research contributes to expanding the literature on the COVID-19 and to filling the gaps in studies on the effect of this shock on food security and livelihood assets, it has some limitations. The first limitation is that the study is cross-sectional, with information on the target variables collected in May 2020 in southern Iran. Thus, caution should be exercised in generalizing its results to other regions and times. The second limitation is related to the research paradigm. Since the paradigm of the study is quantitative, it is recommended to use a qualitative and quantitative–qualitative approach in future works to obtain more precise results.

## 6. Conclusions

The shocks and stress resulting from the COVID-19 pandemic, including the restrictions imposed on people’s lives, are affecting the livelihood and food security of rural families, increasing their food insecurity and their vulnerability to future shocks. The poor, rural population studied in this paper is experiencing worsening food security (73.04%). During the COVID-19 pandemic, the financial (CV = 0.222) and psychological (CV = 0.228) assets of rural families are being influenced more severely than their food security levels. Attention to sustainable livelihoods and the food security of farmers will help to minimize their vulnerability to the pandemic. The results provide new insight into the relationship between the impacts of the COVID-19 shock and the livelihoods and food security of rural families. Furthermore, the results also show how food security can be improved under the described conditions.

The results will help policymakers implement appropriate measures that help to mitigate the harms caused by COVID-19. Given the role of food in supplying safety and health, both governmental and non-governmental organizations should prioritize the support of rural families, especially their livelihood and psychological assets, to help them cope with the pandemic, which drives food insecurity.

## Figures and Tables

**Table 1:** Analysis of the food security situation in rural households.

Food Security Status Level		Scale Values	Number of Affirmative Responses	
Category	Code		(Out of 10) Households without Children	(Out of 18) Households with Children
Food Secure	0	≤2.32	0	0
			1	1
			2	2
Food Insecure Without Hunger	1	2.32–4.56	3	3
	
			4	5
				6
			5	7
Food Insecure With Hunger, Moderate	2	4.56–6.53	6	8
				9
			7	10
				11
			8	12
Food Insecure With Hunger, Severe	3	≥6.53	9 10	13
				14
				15
				16
				17
				18

**Table 2:** Livelihood assets assessment items.

Asset	Item	Number of Items
Financial assets (FIA)	COVID-19 pandemic has reduced the income and purchasing power of rural households	5
	COVID-19 has pushed up food prices among rural households.	
	COVID-19 pandemic has reduced the employment activities of rural households.	
	COVID-19 as a whole has increased the cost to rural households.	
	If COVID-19 pandemic continues, poverty and inequality in rural society will increase.	
Social assets (SOA)	COVID-19 pandemic has decreased people’s trust in each other.	5
	COVID-19 pandemic has greatly diminished social solidarity	
	During COVID-19 pandemic, I do not trust national media information.	
	COVID-19 pandemic has caused the forgetting of traditions and customs (religious celebrations, etc.).	
	COVID-19 has increased the level of social insecurity (crime).	
Human assets (HUA)	COVID-19 pandemic resulted in the closure of educational institutions (schools and universities)	4
	During COVID-19 pandemic, rural communities do not have adequate access to medical staff (nurses and doctor).	
	Rural communities do not have adequate health information to reduce risk of COVID-19 infection.	
	Rural communities do not have sufficient counseling services to cope with effects of COVID-19 pandemic.	
Physical assets (PHA)	Rural communities do not have adequate access to pharmaceutical items during COVID-19 pandemic.	4
	Rural communities do not have adequate access to the required disinfectants and sanitary detergents (masks, gloves, washing gels).	
	Due to the existing facilities and physical structure, the rural community is not able to fully comply with the principles of quarantine and health needed for the containment of the COVID-19 pandemic.	
	Lack of reliable sources of information on the control and treatment of COVID-19.	
Natural assets (NAA)	During COVID-19 pandemic, part of the agricultural activities (such as fertilization, harvesting, etc.) are delayed.	4
	Many natural and recreational facilities are not used in during COVID-19.	
	Agricultural outputs decreased due to COVID-19 pandemic (impossibility of proper management of farmers).	
	Due to the COVID-19, farmers are reluctant to plan to grow their crops.	
Psychological assets (PSA)	I have a lot of anxiety and worry about getting COVID-1.	4
	During COVID-19 pandemic, social tensions are very high.	
	During COVID-19 pandemic, depression and neurological diseases increase.	
	During COVID-19 pandemic, disappointment can be felt on the faces of farmers	

**Table 3:** Prioritizing the items of household food security.

Rank	CV	SD	Mean	Item
1	0.258	0.478	1.85	Could not afford to eat balanced meals
2	0.272	0.457	1.68	Worried food would run out
3	0.280	0.652	2.32	Could not feed children a balanced meal
4	0.286	0.542	1.89	Few kinds of low-cost food for children
5	0.294	0.457	1.55	Children ever hungry
6	0.304	0.502	1.65	Food bought just did not last
7	0.312	0.578	1.85	You ate less than felt you should
8	0.349	0.578	1.68	Adult(s) cut or skipped meals
9	0.376	0.425	1.13	You lost weight because not enough food
10	0.377	0.657	1.74	Children ever skip meals
11	0.420	0.652	1.55	Cut size of children’s meals
12	0.426	0.704	1.65	Children were not eating enough
13	0.451	0.654	1.45	Adult(s) cut or skipped meals, 3+ months
14	0.461	0.854	1.85	You were hungry but did not eat
15	0.516	0.744	1.44	Adult(s) not eat for whole day
16	0.516	0.785	1.52	Children do not eat for whole day
17	0.582	0.652	1.12	Adult(s) do(es) not eat for whole day, 3+ months
18	0.667	0.657	0.985	Children skip meals, 3+ months

CV: Coefficient of variation; SD: Standard deviation.

**Table 4:** The impacts of COVID-19 on livelihood assets.

Category Effects						CV	SD	Mean	Livelihood Assets
Low		Medium		High					
%	Frequency	%	Frequency	%	Frequency				
23.9	70	41.29	121	34.81	102	0.222	0.840	3.78	FIA
						0.228	0.817	3.57	PSA
						0.229	0.854	3.72	PHA
						0.244	0.822	3.36	NAA
						0.341	1.37	40.01	HUA
						0.426	1.40	3.28	SOA

CV: Coefficient of variation; SD: Standard deviation; FIA: Financial assets; PSA: Psychological assets; PHA: Physical assets; NAA: Natural assets; HUA: Human assets; SOA: Social assets.

**Table 5:** Distribution livelihood assets and food security.

Variable	Category	Food Security Groups				Sum	Chi-Squared	Sig
		Secure	Without Hunger	Moderate	Severe			
Ilivelihood assets	Low	38	45	14	5	102	1.082	0.000
	Medium	30	35	50	6	121		
	High	11	22	10	27	70		
	Sum	79	102	74	38	293		

**Table 6:** Model fitting of ordinal regression.

Model	−2 Log Likelihood	Chi-Squared	df	Sig
Intercept Only	425.635	265.842	6	0.000
Final	487.968			

**Table 7:** The impacts of livelihood assets on food security.

Livelihood Assets	Wald	Estimate	EXP (B)	df	Sig
FIA	9.857	0.076	1.02	1	0.001
PSA	8.587	0.068	1	1	0.001
PHA	7.985	0.055	1.01	1	0.001
HUA	6.684	0.058	1.01	1	0.001
SOA	5.857	0.046	1.03	1	0.098
NAA	4.968	0.039	1.04	1	0.135

Significant at the level of 0.01. FIA: Financial assets; PSA: Psychological assets; PHA: Physical assets; NAA: Natural assets; HUA: Human assets; SOA: Social assets; EXP (B): Exponentiation of the B coefficient.

**Table 8:** Test of parallel lines.

Model	−2 Log Likelihood	Chi-Squared	df	Sig
Null Hypothesis	854.767	-	-	-
General	836.234	15.368	6	0.562

## Data Availability

Data is available upon request.

## References

[1] Udmale P., Pal I., Szabo S., Pramanik M., Large A. (2020). Global food security in the context of COVID-19: A scenario-based exploratory analysis. Prog. Disaster Sci..

[2] Swinnen J., McDermott J. (2020). Covid-19 and Global Food Security.

[3] WHO (2020). Impact of COVID-19 on People’s Livelihoods, their Health and Our Food Systems. https://www.who.int/news/item/13-10-2020-impact-of-covid-19-on-people’s-livelihoods-their-health-and-our-food-systems.

[4] Boyacι-Gündüz C.P., Ibrahim S.A., Wei O.C., Galanakis C.M. (2021). Transformation of the Food Sector: Security and Resilience during the COVID-19 Pandemic. Foods.

[5] Van Bodegom A., Koopmanschap E. (2020). The COVID-19 Pandemic and Climate Change Adaptation.

[6] Tajeri Moghadam M., Zobeidi T., Yazdanpanah M. (2020). Analysis of Preventive Behaviors to cope with the epidemic Corona: A New Approach to Rural Development Success. Space Econ. Rural Dev..

[7] Yazdanpanah M., Abadi B., Komendantova N., Zobeidi T., Sieber S. (2020). Some at Risk for COVID-19 Are Reluctant to Take Precautions, but Others Are Not: A Case From Rural in Southern Iran. Front. Public Health.

[8] Defeyter M., Stretesky P., Reynolds C., Furey S., Long M., Porteous D., Dodd A., Stretesky C., Mann E. (2020). The Impact of Covid-19 on Education and Children’s Services: Food Insecurity and Lived Experience of Students (FILES). https://pure.ulster.ac.uk/en/publications/the-impact-of-covid-19-on-education-and-childrens-services-food-i.

[9] Guido Z., Knudson C., Rhiney K. (2020). Will COVID-19 be one shock too many for smallholder coffee livelihoods?. World Dev..

[10] Barrett C.B., Carter M.R. (2013). The economics of poverty traps and persistent poverty: Empirical and policy implications. J. Dev. Stud..

[11] Okpara U.T., Stringer L.C., Dougill A.J. (2017). Using a novel climate–water conflict vulnerability index to capture double exposures in Lake Chad. Reg. Environ. Chang..

[12] Galanakis C.M. (2020). The food systems in the era of the coronavirus (COVID-19) pandemic crisis. Foods.

[13] Rizou M., Galanakis I.M., Aldawoud T.M., Galanakis C.M. (2020). Safety of foods, food supply chain and environment within the COVID-19 pandemic. Trends Food Sci. Technol..

[14] Galanakis C.M., Rizou M., Aldawoud T.M., Ucak I., Rowan N.J. (2021). Innovations and technology disruptions in the food sector within the COVID-19 pandemic and post-lockdown era. Trends Food Sci. Technol..

[15] Paslakis G., Dimitropoulos G., Katzman D.K. (2021). A call to action to address COVID-19–induced global food insecurity to prevent hunger, malnutrition, and eating pathology. Nutr. Rev..

[16] Laborde D., Martin W., Swinnen J., Vos R. (2020). COVID-19 risks to global food security. Science.

[17] Leddy A.M., Weiser S.D., Palar K., Seligman H. (2020). A conceptual model for understanding the rapid COVID-19–related increase in food insecurity and its impact on health and healthcare. Am. J. Clin. Nutr..

[18] Berchoux T., Watmough G.R., Hutton C.W., Atkinson P.M. (2019). Agricultural shocks and drivers of livelihood precariousness across Indian rural communities. Landsc. Urban Plan..

[19] Gatiso T.T., Ordaz-Németh I., Grimes T., Lormie M., Tweh C., Kühl H.S., Junker J. (2018). The impact of the Ebola virus disease (EVD) epidemic on agricultural production and livelihoods in Liberia. PLoS Negl. Trop. Dis..

[20] Chiwaula L., Waibel H. The role of shocks and risks for the livelihoods of small scale fishing communities of Hadejia-Nguru Wetlands in Nigeria. Proceedings of the German Development Economics Conference.

[21] Chambers R., Conway G. (1992). Sustainable Rural Livelihoods: Practical Concepts for the 21st Century.

[22] Scoones I. (1998). Sustainable Rural Livelihoods: A Framework for Analysis. https://www.ids.ac.uk/publications/sustainable-rural-livelihoods-a-framework-for-analysis/.

[23] Bebbington A. (1999). Capitals and capabilities: A framework for analyzing peasant viability, rural livelihoods and poverty. World Dev..

[24] Mphande F.A. (2016). Infectious Diseases and Rural Livelihood in Developing Countries.

[25] Behzadifar M., Behzadifar M., Abdi S., Malekzadeh R., Salmani M.A., Ghoreishinia G., Falahi E., Mirzaei M., Biranvand N.S., Sayehmiri K. (2016). Prevalence of food insecurity in Iran: A systematic review and meta-analysis. Arch. Iran. Med..

[26] Farzana F.D., Rahman A.S., Sultana S., Raihan M.J., Haque M.A., Waid J.L., Choudhury N., Ahmed T. (2017). Coping strategies related to food insecurity at the household level in Bangladesh. PLoS ONE.

[27] Zhou D., Shah T., Ali S., Ahmad W., Din I.U., Ilyas A. (2019). Factors affecting household food security in rural northern hinterland of Pakistan. J. Saudi Soc. Agric. Sci..

[28] Mhlanga D., Ndhlovu E. (2020). Socio-economic implications of the COVID-19 pandemic on smallholder livelihoods in Zimbabwe. BizEcons Q..

[29] HLPE Report (2020). High Level Panel of Experts on Food Security and Nutrition (HLPE).

[30] Fan S., Teng P., Chew P., Smith G., Copeland L. (2021). Food system resilience and COVID-19–Lessons from the Asian experience. Glob. Food Secur..

[31] El Bilali H., Callenius C., Strassner C., Probst L. (2019). Food and nutrition security and sustainability transitions in food systems. Food Energy Secur..

[32] Pakravan-Charvadeh M.R., Mohammadi-Nasrabadi F., Gholamrezai S., Vatanparast H., Flora C., Nabavi-Pelesaraei A. (2021). The short-term effects of COVID-19 outbreak on dietary diversity and food security status of Iranian households (A case study in Tehran province). J. Clean. Prod..

[33] Elsahoryi N., Al-Sayyed H., Odeh M., McGrattan A., Hammad F. (2020). Effect of Covid-19 on food security: A cross-sectional survey. Clin. Nutr. Espen.

[34] Jackson E.A. (2020). Deconstructing Sustainable Livelihood Framework (SLF) for Equitable Living in Crisis of Global Pandemic.

[35] Guillotreau P., Campling L., Robinson J. (2012). Vulnerability of small island fishery economies to climate and institutional changes. Curr. Opin. Environ. Sustain..

[36] Goh A.H. (2012). A literature review of the gender-differentiated impacts of climate change on women’s and men’s assets and well-being in developing countries. Int. Food Policy Res. Washington, D.C. Inst. Capri Work.

[37] Kuang F., Jin J., He R., Ning J., Wan X. (2020). Farmers’ livelihood risks, livelihood assets and adaptation strategies in Rugao City, China. J. Environ. Manag..

[38] Pandey R., Jha S.K., Alatalo J.M., Archie K.M., Gupta A.K. (2017). Sustainable livelihood framework-based indicators for assessing climate change vulnerability and adaptation for Himalayan communities. Ecol. Indic..

[39] Baffoe G., Matsuda H. (2018). An empirical assessment of rural livelihood assets from gender perspective: Evidence from Ghana. Sustain. Sci..

[40] Mbiba M., Collinson M., Hunter L., Twine W. (2019). Social capital is subordinate to natural capital in buffering rural livelihoods from negative shocks: Insights from rural South Africa. J. Rural Stud..

[41] Berhanu W. (2011). Recurrent shocks, poverty traps and the degradation of pastoralists’ social capital in southern Ethiopia. Afr. J. Agric. Resour. Econ..

[42] Ansell N., Hajdu F., van Blerk L., Robson E. (2016). AIDS-affected young people’s access to livelihood assets: Exploring ‘new variant famine’in rural southern Africa. J. Rural Stud..

[43] Azadi Y., Yazdanpanah M., Forouzani M., Mahmoudi H. (2019). Farmers’ adaptation choices to climate change: A case study of wheat growers in Western Iran. J. Water Clim. Chang..

[44] Delfiyan F., Yazdanpanah M., Forouzani M., Yaghoubi J. (2020). Farmers’ adaptation to drought risk through farm–level decisions: The case of farmers in Dehloran county, Southwest of Iran. Clim. Dev..

[45] Pakmehr S., Yazdanpanah M., Baradaran M. (2020). How collective efficacy makes a difference in responses to water shortage due to climate change in southwest Iran. Land Use Policy.

[46] Shinbrot X., Jones K., Rivera-Castañeda A., López-Báez W., Ojima D. (2019). Smallholder farmer adoption of climate-related adaptation strategies: The importance of vulnerability context, livelihood assets, and climate perceptions. Environ. Manag..

[47] Paseban F. (2020). Can the villages deal with Corona? Donya-e-eqtesad Newspaper. Donya-E-Eqtesad Newsp..

[48] Krejcie R.V., Morgan D.W. (1970). Determining sample size for research activities. Educ. Psychol. Meas..

[49] Savari M., Shokati Amghani M. (2020). Factors influencing farmers’ adaptation strategies in confronting the drought in Iran. Environ. Dev. Sustain..

[50] Bickel G., Andrews A., Klein B., Hall D., Stavrianos M. (1996). Measuring food Security in the United States: A Supplement to the CPS. Nutrition and Food Security in the Food Stamp Program.

[51] Pakravan-Charvadeh M.R., Savari M., Khan H.A., Gholamrezai S., Flora C. (2021). Determinants of household vulnerability to food insecurity during COVID-19 lockdown in a mid-term period in Iran. Public Health Nutr..

[52] Gangadharappa H., Pramod K., Shiva K.H. (2007). Gastric floating drug delivery systems: A review. Indian J. Pharm. Educ. Res..

[53] Kalantari K. (2010). Processing social and economic data analysis using SPSS software. Consult. Eng. Publ..

[54] Shiri N., Faghiri M., Pirmoradi A., Agahi H. (2014). Attitudes of agricultural extension workers towards organic farming in Iran. J. Org. Syst..

[55] Zibran M. (2007). CHI-Squared Test of Independence.

[56] Campbell I. (2007). Chi-squared and Fisher–Irwin tests of two-by-two tables with small sample recommendations. Stat. Med..

[57] Lall R., Campbell M., Walters S., Morgan K., Co-operative M.C. (2002). A review of ordinal regression models applied on health-related quality of life assessments. Stat. Methods Med Res..

[58] Christensen R.H.B. (2015). ordinal—regression models for ordinal data. R Package Version.

[59] Abreu M.N.S., Siqueira A.L., Cardoso C.S., Caiaffa W.T. (2008). Ordinal logistic regression models: Application in quality of life studies. Cad. Saúde Pública.

[60] Gutiérrez P.A., Perez-Ortiz M., Sanchez-Monedero J., Fernandez-Navarro F., Hervas-Martinez C. (2015). Ordinal regression methods: Survey and experimental study. IEEE Trans. Knowl. Data Eng..

[61] Savari M., Sheykhi H., Amghani M.S. (2020). The role of educational channels in the motivating of rural women to improve household food security. One Health.

[62] Workie E., Mackolil J., Nyika J., Ramadas S. (2020). Deciphering the impact of COVID-19 pandemic on food security, agriculture, and livelihoods: A review of the evidence from developing countries. Curr. Res. Environ. Sustain..

[63] Khan K.A., Haq M.I., Khan J.M., Zahoor M., Gohar O., Sher M.H., Hameed M.S., Khaliq M.A., Ali S., Kamran A. (2020). Opinion on Impact of Covid-19 Lockdown on Agriculture, Food Security and livelihoods in Pakistan. Int. J. Agric. Biol. Sci..

[64] Ouko K.O., Gwada R.O., Alworah G.O., Onganga Z.M., Ochieng S.V., Ogola J.R.O. (2020). Effects of Covid-19 pandemic on food security and household livelihoods in Kenya. Rev. Agric. Appl. Econ. (RAAE).

[65] Chiwona-Karltun L., Amuakwa-Mensah F., Wamala-Larsson C., Amuakwa-Mensah S., Hatab A.A., Made N., Taremwa N.K., Melyoki L., Rutashobya L.K., Madonsela T. (2021). COVID-19: From health crises to food security anxiety and policy implications. Ambio.

[66] Galanakis C.M., Aldawoud T., Rizou M., Rowan N.J., Ibrahim S.A. (2020). Food ingredients and active compounds against the Coronavirus disease (COVID-19) pandemic: A comprehensive review. Foods.

[67] Dev S.M. (2020). Addressing COVID-19 impacts on agriculture, food security, and livelihoods in India. IFPRI Book Chapters. COVID-19 and Global Food Security.

[68] Kusumawati R.N., Wardani K.K., Suntoro S. (2021). The Psychological State of Farmers in the Agricultural Cultivation of Food Crops during the COVID-19 Pandemic in Java, Indonesia. Caraka Tani J. Sustain. Agric..

[69] Himelein K., Testaverde M., Turay A., Turay S. (2015). The Socio-Economic Impacts of Ebola in Sierra Leone: Results from a High Frequency Cell Phone Survey, Round 3.

